# Intramuscular myxoid lipoma in the proximal forearm presenting as an olecranon mass with superficial radial nerve palsy: a case report

**DOI:** 10.1186/1752-1947-5-321

**Published:** 2011-07-20

**Authors:** Peter Lewkonia, Shaun AC Medlicott, Kevin A Hildebrand

**Affiliations:** 1 Division of Orthopedic Surgery, Department of Surgery, University of Calgary, NW Calgary, Alberta T2N 4Z6, Canada; 2 Department of Pathology & Laboratory Medicine, University of Calgary, NW Calgary, Alberta T2N 4Z6, Canada

## Abstract

**Background:**

Extremity lipomas may occur in any location, including the proximal forearm. We describe a case of a patient with an intramuscular lipoma presenting as an unusual posterior elbow mass.

**Case presentation:**

We discuss the case of a 57-year-old Caucasian man who presented with a tender, posterior elbow mass initially diagnosed as chronic olecranon bursitis. A minor sensory disturbance in the distribution of the superficial radial nerve was initially thought to be unrelated, but was likely caused by mass effect from the lipoma. No pre-operative advanced imaging was obtained because the diagnosis was felt to have already been made. At the time of surgery, a fatty mass originating in the volar forearm muscles was found to have breached the dorsal forearm fascia and displaced the olecranon bursa. Tissue diagnosis was made by histopathology as a myxoid lipoma with no aggressive features. Post-operative recovery was uneventful.

**Conclusion:**

We present a case of an unusual elbow mass presenting with symptoms consistent with chronic olecranon bursitis, a relatively common condition. The only unexplained pre-operative finding was the non-specific finding of a transient superficial radial nerve deficit. We remind clinicians to be cautious when diagnosing soft tissue masses in the extremities when unexplained physical findings are present.

## Background

Nerve entrapment at the elbow has been described affecting the median, ulnar and radial nerves as well as their divisions. Symptoms are variable and depend on the location of the lesion and cause of entrapment. Over the past three decades, radial tunnel syndrome has come to be recognized as a true clinical entity and surgical treatment has become more common [1]. One of the rare causes of compression is a mass lesion such as a parosteal lipoma which may affect either the radial nerve [2], the superficial sensory radial nerve [3] or the posterior interosseous nerve [4]. In these cases, the diagnosis is often delayed and may be confused with lateral epicondylitis or other more common elbow pathologies [5].

Olecranon bursitis is a more common elbow pathology in the general population which may be either acute or chronic, and in the acute setting may be septic or aseptic. Chronic aseptic bursitis is rarely treated surgically, but some cases which are resistant to non-surgical therapies may require more aggressive treatment with bursectomy, with or without bony debridement [6]. Pathology in the olecranon bursa is not expected to cause compression of the radial nerve or related symptoms due to the distance between the two structures.

We present here a case in which an intramuscular myxoid lipoma presented like a chronic aseptic olecranon bursitis that had failed almost 10 years of conservative therapy. The diagnosis was made at the time of surgery. No advanced imaging or further investigation were obtained pre-operatively. Our patient did describe intermittent paresthesia over the distribution of the superficial radial nerve. This finding was not well explained pre-operatively.

## Case presentation

A 57-year-old Caucasian right hand dominant man presented with a 10-year history of a progressively enlarging and mildly tender mass over the bony olecranon of his right elbow. He had initially noted the mass without any previous trauma or pain, and felt the mass was relatively stable in size. During one acute episode after a fall about three years prior to presentation, the mass had seemed to become larger and more bothersome, but was not investigated further. Over the past two years, the mass had been somewhat variable in size, but continued to enlarge slowly. More recently, it had also become mildly tender and particularly uncomfortable when the elbow was placed on a table or desk. He did not have a history of gout or inflammatory arthropathy.

No imaging studies were obtained. Our patient's family doctor made the diagnosis of chronic olecranon bursitis. Two corticosteroid injections were attempted without any change in symptoms. Eventually, a referral was made to an orthopedic elbow surgeon. The first examination by the surgeon revealed a vague mass over the olecranon, measuring approximately 3 × 3 cm. There were no skin changes, and no excessive warmth or erythema to suggest infection. A neurovascular examination demonstrated some decreased sensation to pinprick over the dorsal first web space of the affected hand, with full motor power in the distribution of all major nerves including the radial and posterior interosseous nerves. No other sensory deficits were identified.

Our patient chose to continue observing the mass. At a follow-up appointment about six months later, the decision was made to proceed with surgical treatment with excision of the olecranon bursa for presumed chronic aseptic olecranon bursitis. At that time, the sensory deficit in the hand had almost completely resolved. No further imaging or investigation were pursued.

At the time of surgery, a standard posterior approach to the olecranon bursa was employed. Upon dissection through the subcutaneous tissues, the olecranon bursa was identified but was found to be displaced proximally and superficially by an encapsulated mass extending from the fascia to the lateral side of his proximal dorsal ulna. The capsule was fibrous and easily separated from the subcutaneous tissue although it was inseparable from his dorsal forearm fascia. A yellow, uniform fatty mass was found to be protruding through a defect in his dorsal forearm fascia originating within the most proximal portion of the extensor group. There was no invasion of the local muscle tissue and his elbow capsule was intact and uninvolved.

**Figure 1:**
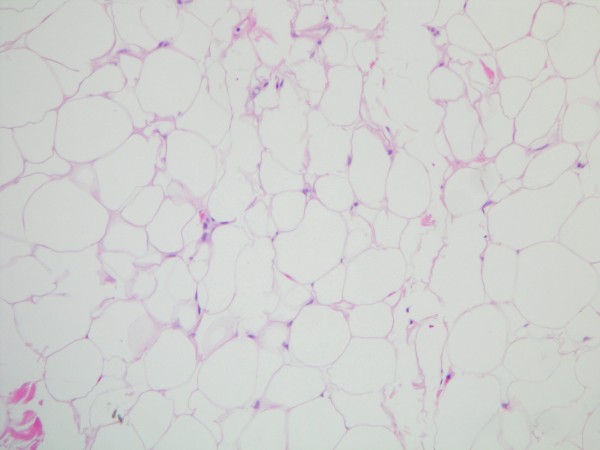
**High-power (200×) H&E stain of benign lipoma tissue from the surgical specimen**. Typical adipocytes with no evidence of malignant change.

**Figure 2:**
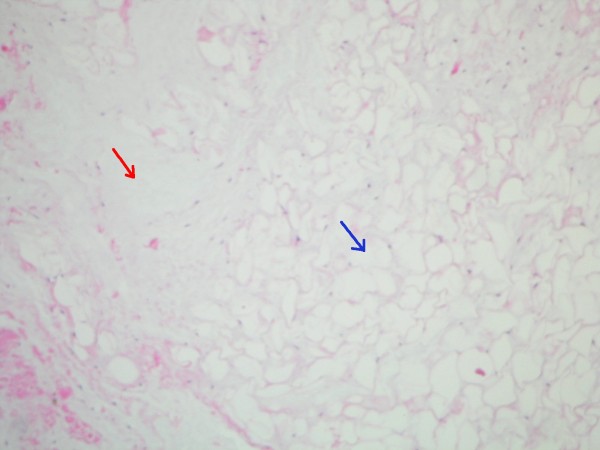
**High-power (100×) H&E stain of myxoid degeneration within benign lipoma**. Adipocytes (blue arrow) interspersed with deposition of mucin-like tissue (red arrow).

Histopathological examination confirmed the diagnosis of a lipoma (Figure 1) with myxoid change (Figure 2) and no evidence of malignancy. Post-operative examination confirmed a normal neurovascular examination with no recurrence of paresthesia and normal power in all muscle groups in his upper extremity. Our patient maintained a full range of motion of his elbow and forearm.

## Discussion

There were several unusual features of this case compared to other reports of proximal forearm or peri-articular lipomas. The neurologic symptoms were intermittent and not considered pre-operatively to be related to the posterior elbow mass. There are several described cases of parosteal or intramuscular lipomas in a similar location, presenting with moderate to severe elbow pain and well defined symptoms affecting either the superficial radial nerve [3] or posterior interosseous nerve [4]. In these cases, the mass itself was contained within a fascial covering. Presumably, the mass effect became more pronounced over time as the slow-growing lesion increased the pressure on the adjacent nerve. With the case presented here, the fascia was violated. This structural abnormality may have prevented increasing pressure on the superficial radial nerve, and instead resulted in a moderately bothersome posterior elbow mass.

In the clinical setting, olecranon bursitis is a common and benign diagnosis that has few specific features to accurately distinguish it from any other soft tissue mass. Both bursitis and non-aggressive soft tissue masses may be present with minimal symptoms for many years before presenting to a surgeon after having failed conservative or non-surgical therapy. This case reminds both primary health care providers as well as specialists that in cases where unusual clinical findings are also present, further imaging or other investigation may be necessary to make appropriate treatment or surgical plans.

## Conclusions

To the best of our knowledge, this is the first report of a lipoma extending through the posterior fascia covering the extensor compartment presenting as a posterior elbow mass. In this case, the unexplained numbness in the distribution of the superficial radial nerve could very plausibly have been caused by a compressive neuropathy by the lipoma as the nerve passed radial to the brachioradialis.

## Consent

Written informed consent was obtained from the patient for publication of this case report and any accompanying images. A copy of the written consent is available for review by the Editor-in-Chief of this journal.

## Competing interests

The authors declare that they have no competing interests.

## Authors' contributions

PL reviewed the available literature, and drafted the manuscript. SM provided the microscope images and provided background information regarding myxoid lipomas. KH assisted with critical review of the manuscript. All authors read and approved the final manuscript.
